# Cognitive Model of Trust Dynamics Predicts Human Behavior within and between Two Games of Strategic Interaction with Computerized Confederate Agents

**DOI:** 10.3389/fpsyg.2016.00049

**Published:** 2016-02-12

**Authors:** Michael G. Collins, Ion Juvina, Kevin A. Gluck

**Affiliations:** ^1^Air Force Research LaboratoryDayton, OH, USA; ^2^Adaptive Strategic Thinking and Executive Control of Cognition and Affect, Department of Psychology, Wright State UniversityDayton, OH, USA

**Keywords:** cognitive modeling, *a priori* model prediction, strategic interaction, trust dynamics, transfer of learning, trust, social dilemma

## Abstract

When playing games of strategic interaction, such as iterated Prisoner's Dilemma and iterated Chicken Game, people exhibit specific within-game learning (e.g., learning a game's optimal outcome) as well as transfer of learning between games (e.g., a game's optimal outcome occurring at a higher proportion when played after another game). The reciprocal trust players develop during the first game is thought to mediate transfer of learning effects. Recently, a computational cognitive model using a novel trust mechanism has been shown to account for human behavior in both games, including the transfer between games. We present the results of a study in which we evaluate the model's *a priori* predictions of human learning and transfer in 16 different conditions. The model's predictive validity is compared against five model variants that lacked a trust mechanism. The results suggest that a trust mechanism is necessary to explain human behavior across multiple conditions, even when a human plays against a non-human agent. The addition of a trust mechanism to the other learning mechanisms within the cognitive architecture, such as sequence learning, instance-based learning, and utility learning, leads to better prediction of the empirical data. It is argued that computational cognitive modeling is a useful tool for studying trust development, calibration, and repair.

## Introduction

Social situations such as business negotiations and conflict resolutions are characterized by a strong interdependence between all parties involved. It has been proposed that people use trust to inform their decisions within these complex and changing environments and a large body of empirical research has been devoted to the topic (e.g., Berg et al., 1995; Mayer et al., 1995; Yamagishi et al., 2005). Trust has been identified as associated with, yet distinct from, other concepts such as cooperation and risk taking (Kee and Knox, 1970). Trust may involve elements of risk taking in the form of “willingness to be vulnerable” (Mayer et al., 1995, p. 712), and trust may lead to cooperation, yet both the former and the latter can exist in the absence of trust. Yamagishi et al. (2005) had participants play a modified version of the game Iterated Prisoner's Dilemma (PD) and observed that cooperation between players emerged at the beginning of an interaction when trust was low and increased as trust developed between participants. Participants were also observed taking small, calculated risks, increasing their trust accordingly, before larger risks were taken. These findings corroborate the claim made by Mayer et al. that trust is distinct from, albeit related to, cooperation and risk taking.

An important distinction in the literature on trust is that between dispositional trust (also referred to as trust propensity) and situational trust (also referred to as learned trust). Berg et al. (1995) found that trust informs the decisions of individuals at the beginning of an interaction. Even with no prior information and no interaction with the other person, participants would trust another player with the expectation that their trust will be reciprocated. Individual differences in one's general willingness to trust another person (i.e., trust propensity) have been identified, finding that some are more willing to trust another without any prior experience (i.e., high trustors) than others (i.e., low trustors) and that these differences lead people to make different choices when beginning to interact with others (Yamagishi et al., 1999).

Situational trust emerges from interaction and consolidates through repeated interaction. When participants played the game iterated Prisoner's Dilemma and were randomly reassigned to a new partner after every round, their trust for the other player was lower compared to when participants were paired with a single partner with whom they played repeatedly, in which case trust increased over time (Yamagishi et al., 2005). Repeatedly interacting with the same person gives an individual the ability to calibrate their trust based on their previous experience with that person. However, the effect of positive and negative events on an individual's trust depends on when the event occurs. Lount et al. (2008) found that negative events that occurred at the beginning of an interaction decreased one's trust in another more so than negative events that occurred later in an interaction. This suggests that an individual's trust is more susceptible to change at the beginning of an interaction when there is little previous experience on which to base one's trust compared to later in the interaction when more experience has been accumulated.

Trust is commonly defined as “the willingness of a party to be vulnerable to the actions of another party based on the expectation that the other will perform a particular action important to the trustor, irrespective of the ability to monitor or control the other party ” (Mayer et al., 1995, p. 712). Although this definition is widely cited, some of its implications are underrepresented in the current theoretical and empirical work on trust. A large amount of effort is dedicated to studying how trust is shaped by the trustor's propensity to trust and the trustee's trustworthiness. We consider that the existing literature addresses mainly the *reactive* component of trust (as derived from the trustor's propensity to trust and the trustee's trustworthiness) and propose here that the *proactive* component of trust (i.e., the trust that the trustee will become trustworthy) is important as well, particularly in understanding trust calibration and trust repair. We believe that the proactive facet of trust is captured in the following elements of the definition presented above (Mayer et al., 1995): “willingness of a party,” “important to the trustor,” and “irrespective of the ability to monitor or control the other party.” One can imagine a situation in which a trustor who has low trust propensity must interact with someone who has shown low trustworthiness. A reactive trust model would predict no trust or even distrust in this situation. However, a proactive trust model would predict at least an attempt to establish trust, based on the assumption that the trustee's trustworthiness may change in the future. We propose here that a trust model that includes both reactive and proactive elements is a more accurate depiction of trust dynamics.

In addition, there is a need to understand how different factors, which have been found to affect trust, interact with each other to explain the choices made by individuals over time. One approach to gain such insight is to use computational cognitive models of how trust is used in specific situations. Developing models within a cognitive architecture such as ACT-R (Adaptive-Control of Thought—Rational; Anderson, 2007), Soar (Laird, 2012), or EPIC (Kieras and Meyer, 1997) allows for a formalized theory of trust to be implemented using general cognitive mechanisms. Cognitive models also have the ability to be run in the same experiments as participants, allowing for direct comparison of human and model data. This approach supports the development and evaluation of substantive, mechanistic cognitive theory.

Recently, a computational cognitive model using a novel trust mechanism has been shown to account for human behavior in both iterated Prisoner's Dilemma (PD) and iterated Chicken Game (CG), including the transfer of learning between the two games (Juvina et al., 2015). Here, we examine the predictive validity of this model. We present the results of a study in which the model's a priori predictions of behavior in 16 different conditions are compared to the behavior of human participants. To anticipate, the results show that a trust mechanism is necessary to explain human behavior across multiple conditions, even when a human plays with a non-human agent.

### Games

**Figure 1:**
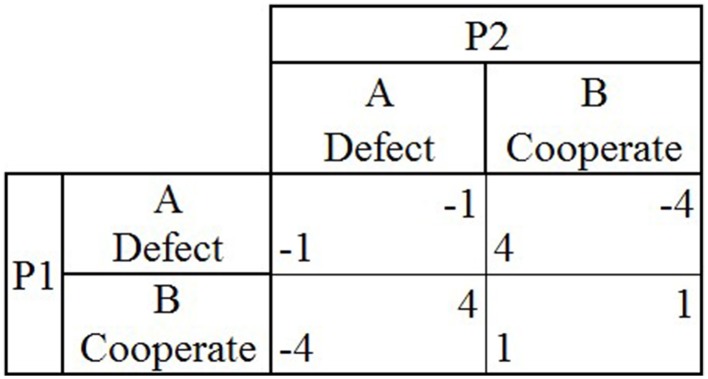
**The payoff matrix for the game Prisoner's Dilemma shows the two players (P1 and P2), the two possible choices they can make during each round (A or B) and the four possible outcomes that can occur during a single round (P1's payoff located in lower left hand corner, P2's payoff in upper right hand corner) depending on the choices made by both players in that round**.

Games of strategic interaction are often used to study how people behave in different situations. A game represents an abstraction of a real world scenario, where the outcomes that occur are determined by the choices made by all of the players. One of the simplest types of games of strategic interaction are 2 × 2 games, which consists of two players choosing to either cooperate or defect (B and A) leading to one of four possible outcomes after both players make a choice. The payoffs for each player associated with each outcome within a game vary, in turn affecting the behavior of both players. Two commonly used games in both psychological and economic research are iterated Prisoners Dilemma (PD) and iterated Chicken Game (CG). In PD, if both players choose to cooperate (B) (mutual cooperation), each earns one point. If both players choose to defect (A), then each loses one point. If one player chooses to defect (A) and the other chooses to cooperate (B), the player who chose to defect (A) earns four points, while the other player loses four points (Figure 1). When PD is played repeatedly and each player does not know when the game will end, the optimal strategy is for both players to choose to repeatedly cooperate (B), earning one point during each round.

**Figure 2:**
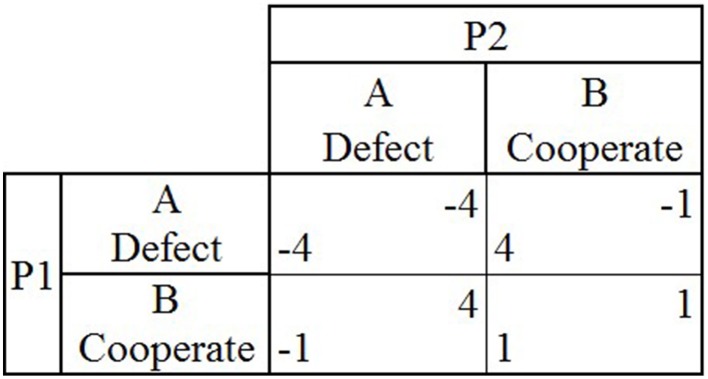
**The payoff matrix for the game Chicken shows the two players (P1 and P2), the two possible choices they can make during each round (A or B) and the four possible outcomes that can occur during a single round (P1's payoff is located in lower left hand corner and P2's payoff is located in upper right hand corner) depending on the choices made by both players in that round**.

In CG, both players again have the option to either defect or cooperate (A or B; Figure 2). If both players choose to cooperate (B), then each player earns one point, as in PD. If both players choose to defect (A), then both lose four points. If one player chooses to defect (A) and the other player chooses to cooperate (B), the player who chose to cooperate (B) loses one point and the player who chose to defect (A) earns four points. When CG is played repeatedly and both players do not know when the game will end, the optimal outcome is for players to repeatedly asymmetrically alternate between choosing to cooperate and defect (B and A), to earn 1.5 points each during each round.

Although the payoffs associated with each choice in PD and CG are different, certain similarities exist between the two games. The first is a surface similarity, represented by a similarity in the payoff matrix of each game, which is that during both PD and CG players can both choose B and each earn one point. The second is a deep similarity, not represented on the payoff matrix but in abstract meaning. The deep similarity between PD and CG is that the optimal outcome in each game (i.e., repeated mutual cooperation in PD and asymmetric alternation in CG) requires players to mutually cooperate and coordinate their behavior.

### Confederate agents

In the research that led to the development of the trust model (Juvina et al., 2013, 2015), human participants played games in pairs with one another. In the study reported here, human participants were paired with preprogrammed agents, rather than with other humans. The agents used a deterministic strategy with intermittent random choices during a game. The main reason for using preprogrammed agents is that it allows us to manipulate trustworthiness and expand the range of conditions in which the model is tested. Two deterministic strategies that have been used in prior research are the Tit-for-Tat strategy (T4T; Axelrod, 1984) and the Pavlov-Tit-for-Tat (PT4T; introduced in Juvina et al., 2012). T4T repeats on round *N* the other player's previous choice from round *N* – 1 (Table 1). The PT4T strategy is a combination of two strategies, T4T and Pavlov (Nowak and Sigmund, 1993). Pavlov is another deterministic strategy that operates under the rule of win-stay-lose-shift, only switching choices at round *N* if it lost points with its previous choice at round *N* – 1 (Table 2). Although both the T4T and Pavlov strategy have shown to be competitive strategies during repeated computer simulations (Nowak and Sigmund, 1993), Juvina et al. (2012) found that the repetition propensities (i.e., the probability that an individual will repeat the same choice following a particular outcome) of the T4T or Pavlov strategy did not match the repetition propensity of humans during PD. When playing PD, humans were found to be less likely to switch choices after an outcome in which they earned points while the other player lost points, unlike the T4T strategy which immediately changes its choice on the next round. To create a strategy that had a more human like repetition propensity, the win-stay component of Pavlov was combined with the T4T strategy to make the PT4T strategy (Table 3). PT4T reciprocates the other player's choice after mutual cooperation and mutual defection and switches choices after unilateral cooperation (i.e., the strategy chose B and the other player chose A). After instances of unilateral defection (i.e., the strategy chose A and the other player chose B), the PT4T strategy will again choose to defect (i.e., win-stay; see Tables 1, 2).

**Table 1:** **Choices that the Tit-For-Tat (T4T) strategy would make on round *N* during both Prisoner's Dilemma (PD) and Chicken Game (CG) based on the previous choice (round *N* – 1) of both the strategy and other player**.

**Tit-For-Tat's strategic choices**				
**Game**	**Round** ***N*** **– 1**			**Round** ***N***
	**T4T's choice**	**Other player's choice**	**T4T's payoff**	**T4T's choice**
PD	A	A	−1	A
PD	A	B	4	B
PD	B	A	−4	A
PD	B	B	1	B
CG	A	A	−4	A
CG	A	B	4	B
CG	B	A	−1	A
CG	B	B	1	B

**Table 2:** **Choices that the Pavlov strategy would make on round *N* during both Prisoner's Dilemma (PD) and Chicken Game (CG) based on the previous choice (round *N* – 1) of both the strategy and the other player**.

**Pavlov's strategic choices**				
**Game**	**Round** ***N*** **– 1**			**Round** ***N***
	**Pavlov's choice**	**Other player's choice**	**Pavlov's payoff**	**Pavlov's choice**
PD	A	A	−1	B
PD	A	B	4	A
PD	B	A	−4	A
PD	B	B	1	B
CG	A	A	−4	B
CG	A	B	4	A
CG	B	A	−1	A
CG	B	B	1	B

**Table 3:** **Choices that the Pavlov-Tit-For-Tat (PT4T) strategy would make on round *N* during both Prisoner's Dilemma (PD) and Chicken Game (CG) based on the previous choice (round *N* – 1) of both the strategy and other player**.

**Pavlov–Tit-For-Tat's strategic choices**				
**Game**	**Round** ***N*** **– 1**			**Round** ***N***
	**PT4T's choice**	**Other player's choice**	**PT4T's payoff**	**PT4T's choice**
PD	A	A	−1	A
PD	A	B	4	A
PD	B	A	−4	A
PD	B	B	1	B
CG	A	A	−4	A
CG	A	B	4	A
CG	B	A	−1	A
CG	B	B	1	B

### Transfer of learning

A game represents a single situation in which individuals interact, but in the real world people might mix with a single person across a variety of situations, using what they have learned about the behavior of another in one context to inform their decisions in a new situation. For this reason it is often of interest to examine how individuals behave when interacting across different games. When PD and CG are played sequentially, specific transfer of learning effects occur, such that the outcomes during the first game affect the outcomes during the second game (Juvina et al., 2013). For example, a higher proportion of asymmetric alternation is observed during CG when played after PD and a higher proportion of mutual cooperation is seen during PD when played after CG. Previous explanations for these transfer effects, such as a similarity between the games, the expectation the other player will behave as they did in the past, or a strategy used during a simpler game continuing to be used in a more complex game, were unable to account for all of the transfer effects that were seen when PD and CG were played in either order (Knez and Camerer, 2000; Devetag, 2005; Bednar et al., 2012). To account for the transfer of learning effects observed between PD and CG, Juvina et al. (2013) proposed that deep transfer effects are mediated by the reciprocal trust between players during games of strategic interaction. This explanation of transfer effects has since been implemented as an ACT-R model (details later in the paper). The model accounts for human behavior without using a predetermined strategy, but instead using several general learning mechanisms and a novel trust mechanism which influences its choices over the course of both games (Juvina et al., 2015).

### Experiment

This experiment is intended to test the extent to which the model from Juvina et al. (2015) can generalize to a different sample of participants playing with computer agents who used preprogrammed strategies. We went beyond model fitting and adopted the challenging practice of using an existing model to predict the behavior of participants in a new experiment to assess the model's predictive validity. The model from Juvina et al. (2015) was used to generate *a priori* predictions of the behavior of participants when playing the games PD and CG in varying orders with a confederate agent who used either the T4T or PT4T strategy and whose trustworthiness was manipulated. Several changes were introduced into the experimental design from Juvina et al. (2013), such as a decrease in the number of rounds per game, different payoff matrices, performance-based incentives, two new game orders, and the specific strategy that participants played with, challenging the model's ability to account for human behavior under these new conditions. In addition, we introduced a manipulation check to verify that each participant's trust was sensitive to the manipulation of the confederate agent's trustworthiness. Two trust questionnaires were used as a manipulation check: trait trust, also referred to as trust propensity, is a measure of an individual's general dispositional trust toward others; state trust, also referred to as learned trust or situational trust, refers to the specific trust that an individual has for a particular person in a particular situation, in this case the participant's trust in the confederate agent.

All of the model's predictions were made publically available in April of 2015 on http://psych-scholar.wright.edu/ijuvina/publications. The predictions were then published in the proceedings of the 10th annual conference on Behavioral Representation in Modeling and Simulation (Collins et al., 2015). In this paper, the model's predictions are compared to the behavior of human participants. The model's predictions across all conditions are also compared to five alternative variants, of the model which did not use a trust-mechanism to account for the behavior of participants.

### Hypotheses

The goal of this experiment was to examine the predictive validity of an existing model, thus the *a priori* predictions generated by this model across all conditions serve as hypotheses. In addition, three specific hypotheses about the participants' self-reported trust and the model's trust mechanism were made. First, because the participant's reported trait trust (i.e., trust propensity) is a generalized disposition toward trusting others, we predict that (1a) participants' trait trust will not change over the course of the experiment and (1b) will not significantly predict the participants' behavior during the experiment. Second, because a participant's reported state trust is specific to the other player, we predict that (2a) the participants' state trust will be sensitive to the strategy and trustworthiness of the confederate agent and (2b) their state trust after the first game will be a significant predictor of their behavior during the second game. Third, based on the model predictions, we hypothesize that (3a) no deep transfer effects between games will occur in any of the conditions, (3b) the model will account for the behavior of human participants better than any of the other model variants, and (3c) each component of the model's trust mechanism will be required to account for human behavior.

## Methods

### Participants

For this study, 320 participants (176 male, 144 female; *M*_*age*_ = 36.62, *SD*_*age*_ = 10.67) were recruited from the website Amazon Mechanical Turk (AMT). AMT is an online labor market where individuals (workers) complete brief tasks, human intelligence tasks (HIT) as they are referred to on AMT, in exchange for a predetermined amount of money. To be eligible to participate in the experiment, workers had to have completed a minimum 10,000 other HITs and have an approval rating of 95% or higher. The reason we required a minimum number of HITs by workers was to ensure data quality. Peer et al. (2014) found that workers with high experience (i.e., those who completed more HITS) followed instructions and replicated known effects better than workers who had completed fewer HITS. All participants received a base pay of $1 for completing the study and had the opportunity to earn up to $2 extra dollars based on the cumulative number of points they earned during both games.

### Design

Each participant played two games of strategic interaction (i.e., PD Figure 1 and CG Figure 2) for 50 rounds each, playing either game once (PDCG or CGPD order) or one game twice (PDPD or CGCG order). Participants played with a confederate agent whose strategy and trustworthiness were manipulated throughout and held constant over the course of both games (Table 4). Each participant was told that the confederate agent was another individual recruited from AMT, with whom they would play throughout both games. The participants were debriefed afterwards about the identity of the confederate agent. The study was approved by the Write State University's Institutional Review Board.

**Table 4:** **The 16 conditions to which participants were randomly assigned during the experiment**.

**Experimental conditions**				
**Condition**	**Game order**		**Confederate agent**	
	**Game 1**	**Game 2**	**Strategy**	**Trustworthiness**
1	PD	PD	T4T	HT
2	PD	CG	T4T	HT
3	CG	PD	T4T	HT
4	CG	CG	T4T	HT
5	PD	PD	PT4T	HT
6	PD	CG	PT4T	HT
7	CG	PD	PT4T	HT
8	CG	CG	PT4T	HT
9	PD	PD	T4T	LT
10	PD	CG	T4T	LT
11	CG	PD	T4T	LT
12	CG	CG	T4T	LT
13	PD	PD	PT4T	LT
14	PD	CG	PT4T	LT
15	CG	PD	PT4T	LT
16	CG	CG	PT4T	LT

They played a combination of two games, Prisoner's Dilemma (PD), and Chicken Game (CG), or the same game twice. During each condition participants played games with a confederate agent who used either the Tit-for-Tat (T4T) or Pavlov-Tit-for-Tat (PT4T) strategy and was either high (HT) or low (LT) in trustworthiness.

### Confederate agent

The confederate agent utilized one of two predetermined strategies, T4T or PT4T. The T4T strategy chose on round *N* the same choice that the other player made on round *N* – 1 (Table 1). The PT4T strategy reciprocated mutual cooperation, mutual defection, and unilateral defection (as T4T), but did not reciprocate unilateral cooperation (as Pavlov; see Table 2). Along with using one of two predetermined strategies, the confederate agent's trustworthiness was manipulated. In addition, randomness was added into the agents' behavior to make it less deterministic and to mimic human-like behavior. To manipulate trustworthiness, the confederate agent either randomly cooperated or randomly defected a certain number of times over the course of each game. In the high trustworthiness (HT) condition the confederate agent randomly cooperated and in the low trustworthiness (LT) condition the confederate agent randomly defected. The number of times that the confederate agent randomly cooperated or randomly defected was determined in advance. Multiple simulations were run before the experiment varying the frequency the confederate agent cooperation or defection and examining the models' predictions. Because PT4T is inherently less trustworthy than T4T (i.e., more apt to exploit the other player's attempts to establish mutual cooperation), to avoid the model predicting a high proportion of mutual defection during both the PT4T HT and the PT4T LT conditions, a larger percentage of cooperation was needed to significantly raise PT4T's trustworthiness, as determined by the model simulations. Thus, during the T4T conditions, the confederate agent would employ the T4T strategy during 90% (total of 45 rounds) of the game, while randomly cooperating (in the HT conditions) or defecting (in the LT conditions) on 10% (total of five rounds) of the rounds. During the PT4T conditions, the confederate agent employed the PT4T strategy during 65% (total of 33 rounds) of the game and randomly cooperated (in the HT condition) or defected (in the LT condition) during 35% (total of 17 rounds) rounds of the game.

### Materials and measurements

All participants took surveys to measure their trait and state trust.

#### Trait trust

The trait trust questionnaire was a 24-item questionnaire (Appendix A in Supplementary Material) rated on a five-point Likert scale (1: *lowest*–5: *highest*) and was used to measure a participant's general willingness to trust others (Colquitt et al., 2007). It was administered before the first game and after the second game. The trait trust survey was composed of a combination of questions from Rotter (1967), Yamagishi (1986) trust propensity surveys and several items created by the experimenters.

#### State trust

The state trust questionnaire was a 14-item questionnaire (Appendix B in Supplementary Material) rated on a five-point Likert scale (1: *lowest*–5: *highest*) and was used to measure the participant's specific trust in the confederate agent over the course of the game. It was administered before and after each game. The items used in the state trust questionnaire examined the willingness of the participant to allow the other player to influence the game outcomes and the participant's expectations about the other player and their behavior. The state trust questionnaire was conceived by the experimenters to be congruent with the definition of trust proposed in Mayer et al. (1995).

### The model

Here was present an overview of the model used to generate predictions of participants' behavior across the different conditions. A more detailed description of the model and how it is implemented in the ACT-R architecture can be found in Juvina et al. (2015). ACT-R is a cognitive architecture and a formal theory of human cognition (Anderson, 2007), in which cognition occurs through the interaction of multiple modules, such as the declarative (know-what) and procedural (know-how) memory modules. The declarative memory module stores information that the model has learned from the environment. The procedural memory allows for action selection reinforced through reward patterns that occur within the environment (Anderson, 2007). To account for the behavior of individuals within and between PD and CG, the model uses a novel trust mechanism in addition to the traditional mechanisms of the architecture.

In order for the model to be able to learn to play either game, it needs to be aware of the interdependence between itself and the other player; to do this the model uses instance-based learning (IBL; Gonzalez et al., 2003). In IBL, past instances of an event are stored in a model's declarative memory to be recalled later to inform its future decisions. During each round, the model stores in its declarative memory the previous choice of both itself and the other player along with the other player's choice for the current round. Throughout both games, each time the model stores a copy of a previous instance that has already been placed in its declarative memory it increases the probability that specific instance will be recalled when placed in a similar situation again, as controlled by ACT-R's activation equations (Anderson, 2007).

To account for the behavior of participants without relying on using a predetermined strategy, the model uses IBL, sequence learning, and reinforcement learning, to learn how to play either game. During each round, the model attempts to recall a previous instance from memory using both its own and the other player's previous choices as retrieval cues. The previous instances stored in the model's declarative memory allow it to implement sequence learning, recalling what the other player's next choice was when placed in that situation before. The model then predicts that the other player will choose the move that was chosen most frequently when placed in a similar situation in the past. Next, the model chooses to cooperate or defect depending on which choice has the greatest utility given the model's prediction of the other player's choice. Previous rewards the model has received for cooperating and defecting in similar contexts (i.e., the other player's expected next choice based on the previous choice of the other player and the model) determine the utility or the value of these choices to the model (Juvina et al., 2015).

Using a combination of IBL, sequence learning, and reinforcement learning allows the model to learn how to play either game with another opponent, but these mechanisms alone cannot account for the deep transfer effects seen when the games are played sequentially. In order to account for the deep transfer effects, a novel trust mechanism consisting of two accumulators and three different reward functions was added to the model. The two accumulators are called trust and trust-invest. Each accumulator starts at zero at the start of the first game and increases or decreases depending on the choices made by both the model and the other player after each round. The trust accumulator is used to track the trustworthiness of the other player; it increases after outcomes of mutual cooperation and unilateral defection (i.e., instances where the other player has shown to be trustworthy) and decreases after outcomes of mutual defection and unilateral cooperation (i.e., instances where the other player has shown to be untrustworthy). The trust-invest accumulator is used to track trust necessity, that is, the need to establish trust with the other player (e.g., in cases where both players are continually defecting), it increases after instances of mutual defection and decreases after instances of unilateral cooperation. Throughout the game, the current level of the trust and trust-invest accumulators determines the current reward function used to reinforce the model's choices. This is a principled way of changing the model's behavior over the course of the game based on interaction with the other player.

The three different reward functions used by the model each reward the model differently for each of the four possible outcomes that can occur during a game, causing the model to learn different strategies. When the trust accumulator is positive, regardless of the level of the trust-invest accumulator, the model is reinforced for increasing the payoff of both players minus the other player's previous payoff. When only the trust-invest accumulator is positive, the model is reinforced for increasing the payoff of the other player, signaling the desire to establish a trust relationship with the other player. When both accumulators are at or below zero (i.e., when trust has not been established or has been lost and the repair attempts have failed), the model is reinforced for maximizing its own payoff and minimizing the payoff of the other player. By switching between three different reward functions, the value the model places on different choices changes depending on the previous interaction between the model and the other player, in turn affecting whether the model learns to maximize the payoff of both players, only the other player's payoff, or only its own payoff.

### Model predictions

All of the model's predictions were generated by placing the model in each of the 16 experimental conditions and running it 50 times, playing each game with one of 10 versions, mimicking the procedure that participants followed. To ensure pseudo-random variability of the confederate agent across participants and model, 10 different versions of each combination of the confederate agent's strategy and trustworthiness were created^1^. Once assigned to a condition the model was randomly assigned to play each game with one of the 10 possible versions of the confederate agent, as were the participants. A discussion of the model's predictions can be found in Collins et al. (2015). The only part of the experimental procedure that was not copied by the model was the time between the two games, in which participants took the state trust survey and received instructions for the next game.

### Procedure

Once recruited from Amazon Mechanical Turk, participants were instructed to go to a website where the experiment was located. On that website, participants first gave their informed consent, then completed a demographic questionnaire and the trait trust survey to measure their trust propensity. They were randomly assigned to one of the 16 experimental conditions and then they were randomly assigned to play one of the 10 possible versions of each game in each condition.

Before playing each game, participants received instructions presented in two parts. The first part of the instructions explained the game's payoff matrix. The second part explained how the payoff matrix could be used to make decisions during the game. The instructions stated that the games would be played repeatedly and that points earned during a round depended on the choices made by both players. After reading the game's instructions, participants were required to answer five questions about the game to verify that they understood the instructions. The five questions asked, given the choices made by two hypothetical players, what would the payoff of one of those players be (e.g., If Player 1 chose A and Player 2 chose B, what is Player 1's payoff for that round?). If a participant answered fewer than four of the five questions correctly, they had to reread the instructions until they were able to answer at least four out of five questions correctly.

Once the instructions were completed, participants were notified that they would play the game with another worker who was also recruited from AMT and then went on to play 50 rounds of the first game with the confederate agent. After the first game, participants completed the state trust survey, read the instructions for the second game, and then went on to play another 50 rounds of the second game with the same confederate agent as in the first game. Finally, participants took the state and trait questionnaires again, were debriefed to the true nature of the confederate agent, and received payment for their participation.

## Results

### Manipulation check

Before the behavior of participants was compared to the model's predictions, we first examined the participants' trait and state trust scores to ensure that the experimental manipulations did affect their trust during the experiment. We hypothesized that the experiment would not affect the participants' trait trust, because trait trust is a general disposition to trust others, that is, it would not be specific to the confederate agent and would not be a significant predictor of the participant's behavior. Trait trust was computed by taking the average of the ratings of the trait trust questionnaire's items. The trait trust questionnaire was administered both before and after the experiment.

**Figure 3:**
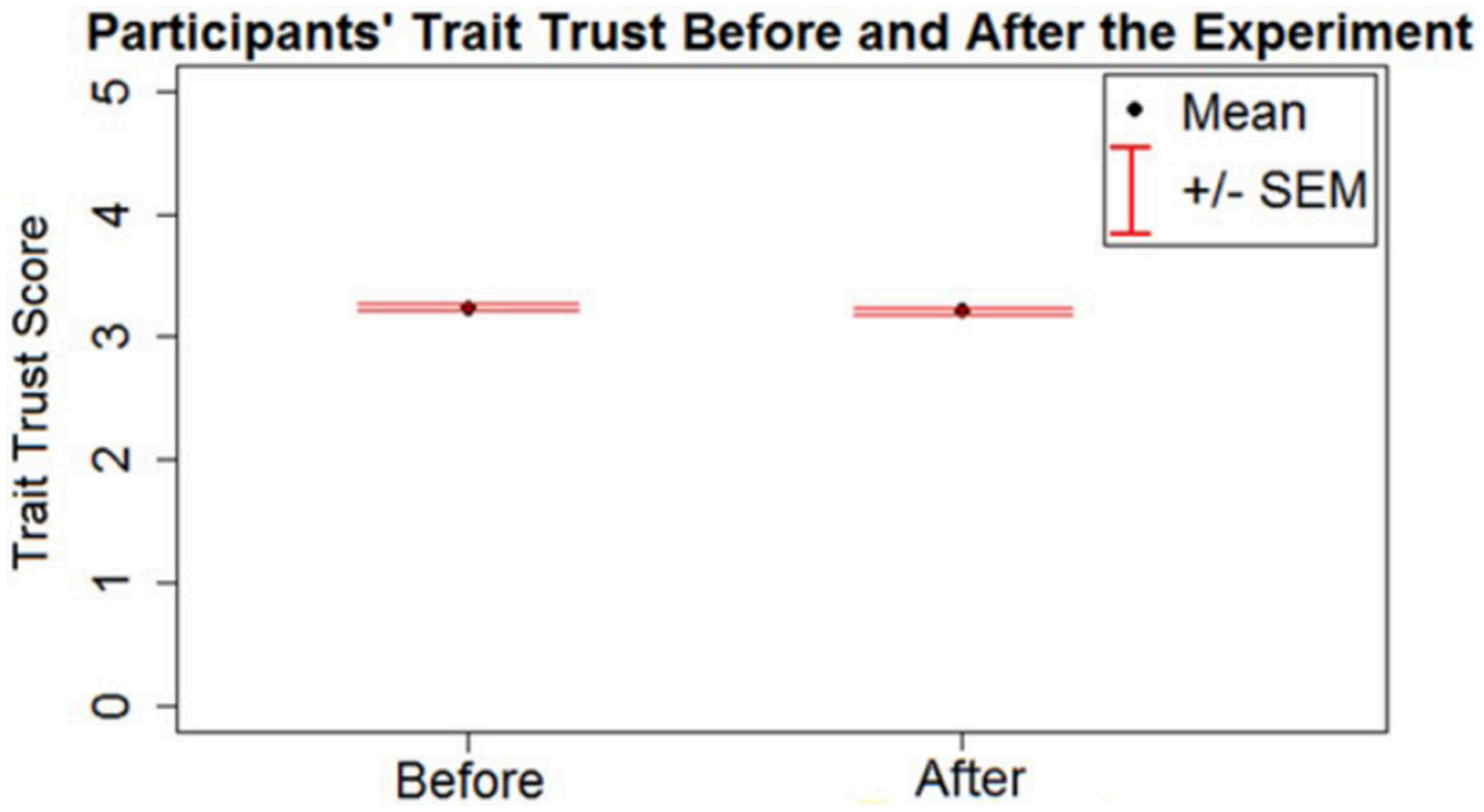
**Trait trust measured before and after the experiment; the error bars represent standard error of the mean**.

To examine whether participants' reported trait trust changed over the course of the experiment, a paired *t*-test was run on trait trust scores before and after the study. The trait trust score before (*M* = 3.23, *SD* = 0.57) and after (*M* = 3.20, *SD* = 0.61) the experiment were found to be statistically different [*t*_(313)_ = 2.08, *p* < 0.05], decreasing slightly after playing the games (Figure 3). To test if the change in trait trust was affected differently by the confederate agent strategies and levels of trustworthiness, an analysis of variance (ANOVA) was run on the difference score (*dif*) of the reported trait trust (i.e., the trait trust before the experiment subtracted from the trait trust after the experiment) as a dependent variable with the strategy and trustworthiness of the confederate agent as factors. The ANOVA found no significant main effect of the confederate agent's strategy (*p* > 0.05), but a significant main effect of the confederate agent's trustworthiness was found [*F*_(1, 310)_ = 6.88, *p* < 0.05]. The participants' trait trust score decreased after playing with a low trustworthiness agent (*M*_*dif*_ = 0.07, *SD*_*dif*_ = 0.33) and increased slightly after playing with a high trustworthiness agent (*M*_*dif*_ = −0.007, *SD*_*dif*_ = 0.22).

Finally, to observe if the participants' reported trait trust predicted their behavior during the experiment, a linear regression was run using a participant's trait trust before the experiment to predict the overall frequency they cooperated in the experiment. The participants' reported trait trust before the experiment was found not to be a significant predictor of their overall frequency of cooperation during the experiment (*p* > 0.05).

With regard to state trust, it was predicted that participants would be sensitive to the trustworthiness and strategy of the confederate agent, thus their trust in the other player would vary across conditions. Based on the model's predictions, we expected that the participants would have less trust in the confederate agents that used the PT4T strategy and had low trustworthiness. Both of these two factors (i.e., the PT4T strategy and low trustworthiness manipulation) make the confederate agent more likely to unilaterally defect and thus appear less trustworthy. The state trust of participants was computed as the average of all of their responses to the state trust survey taken after the first and second game in each condition, averaging these two scores.

**Figure 4:**
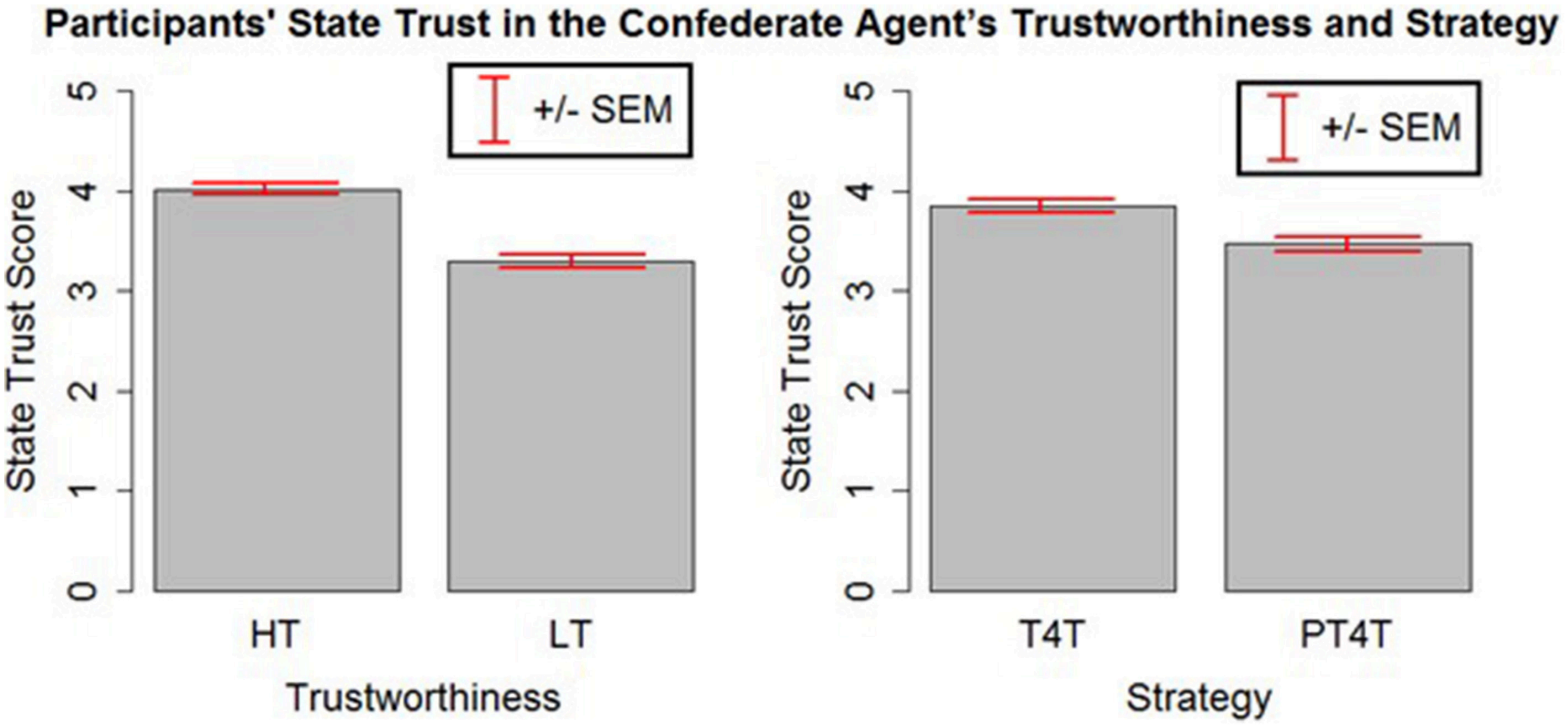
**The mean and standard error of the mean of the participants' state trust**. Participants were found to have more trust in the high (HT) than the low (LT) trustworthiness agent and the Tit-for-Tat (T4T) than the Pavlov-Tit-For-Tat (PT4T) strategy.

To compare the state trust scores of participants across different conditions, an analysis of variance (ANOVA) on the participant's averaged state trust score was run with the confederate agent's strategy and trustworthiness as factors. The ANOVA revealed significant main effects of strategy [*F*_(1, 316)_ = 11.49, *p* < 0.05] and trustworthiness [*F*_(1, 316)_ = 73.182, *p* < 0.05]. As expected, participants were found to have more trust in the confederate agent who used the T4T (*M* = 3.85, *SD* = 0.82) strategy than PT4T (*M* = 3.46, *SD* = 0.94) strategy and the agent whose trustworthiness was high (*M* = 4.02, *SD* = 0.80) compared to low (*M* = 3.29, *SD* = 0.85; Figure 4). This suggests that the experimental manipulations to the confederate agent's strategy and trustworthiness had a significant effect on the participants' trust.

Finally, we examined if the participant's state trust score after the first game significantly predicted the frequency of cooperation during the second game. We hypothesized that, because state trust is specific to the other player, participants with a higher state trust score in the confederate agent will be more willing to cooperate during the second game. A linear regression with the participants' frequency of cooperation during the second game as a dependent variable and their state trust score after the first game as a predictor was run. Although the regression accounted for only 10% of the variance in the data [*R*^2^ = 0.10, *F*_(1, 318)_ = 36.76, *p* < 0.01], the participants' state trust score after the first game was found to significantly predict their frequency of cooperation during the second game (β = 0.05, *p* < 0.01).

### Performance metrics

When comparing the model's predictions to the behavior of human participants across the 16 different conditions, two performance metrics during both games were examined. The first was the proportion of each outcome (i.e., mutual cooperation, unilateral cooperation, unilateral defection, mutual defection, and alternation) during each round across 50 model runs (i.e., simulated participants) and 20 human participants in each condition. Instances of alternation were determined by identifying rounds where the participant (or model) and the confederate agent made opposite choices on round *N* and had each made the opposite moves on round *N* – 1 (for example, moves A and B on round *N*, while moves B and A on round *N* – 1). The round-by-round proportion of alternation was then computed like all other outcomes.

The second performance metric was the round-by-round repetition propensities. The round-by-round repetition propensity (Rapoport, 1967) was calculated by identifying on each round the probability that either the model or participant would repeat the same move after a particular outcome in the previous round (e.g., the probability that a participants who were involved in mutual defection outcome on round 5 would choose to defect again on round 6 is represented as A after AA). The round-by-round repetition propensity was examined instead of the overall average repetition propensity of the model or human participants, because the temporal dynamics of the interaction are lost when the average repetition propensity is taken. We use these two metrics to assess the predictive validity of the model's *a priori* predictions.

### Predictive validity

The main purpose of this experiment was to examine the predictive validity of the model, that is, determine how well a model that was fit to another dataset generalized to a new sample of participants and a new set of experimental conditions. The correlation (*r*) and the root mean squared deviation between the round-by-round proportion of all five outcomes in both the model predictions and human participants were computed, both over all 16 and each individual condition. The overall proportion (i.e., mean of the round-by-round proportion) of each outcome in both the model predictions and human participants were also used to evaluate the model's fit.

**Figure 5:**
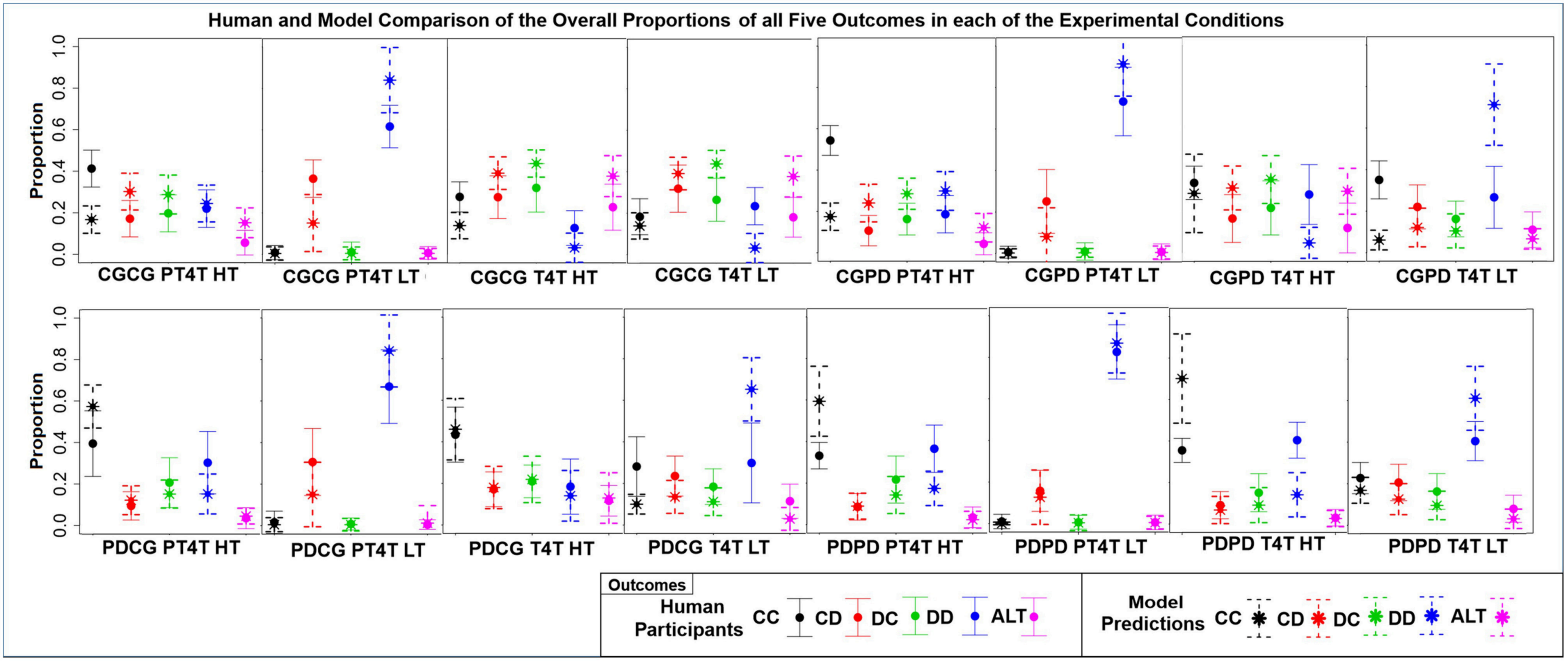
**The mean ± one standard deviation of the overall proportion that each of the five outcomes [mutual cooperation (CC), unilateral cooperation (CD), unilateral defection (DC), mutual defection (DD), and alternation (ALT)] was choosen over the course of both games in each of the 16 conditions, during both the model predictions (star and dashed lines) and human participants (dot and solid lines)**.

The overall *r* and RMSD (*r* = 0.66, *p* > 0.001, *RMSD* = 0.19) across all of the experimental conditions was lower than the model's original fit (*r* = 0.87, *RMSD* = 0.09) to data from Juvina et al. (2013). However, the overall correlation between the model's *a priori* predictions and the current human data is encouraging taking into account the fact that the model was fit to a single other dataset and then used to predict the behavior of individuals across 16 different conditions. From the *r* and RMSD between the model and participants in each condition and the comparison of the overall proportion of each outcome, three observations can be made about the model's predictions (Figure 5; Table 5).

**Table 5:** **Correlation (*r*) and root mean squared deviation (RMSD) between the model's predictions and the human behavior in each condition, averaged across all five outcomes**.

**Correlation and RMSD between the model's predictions and the human participants**					
**Condition**	***r***	**RMSD**	**Condition**	***r***	**RMSD**
CGCG PT4T HT	−0.03	0.18	PDPD T4T HT	0.55	0.23
CGPD PT4T HT	−0.03	0.22	PDCG T4T HT	0.66	0.14
CGPD T4T HT	−0.01	0.22	PDPD T4T LT	0.67	0.17
CGCG T4T LT	0.22	0.19	PDCG PT4T HT	0.68	0.15
CGPD T4T LT	0.28	0.27	CGCG PT4T LT	0.86	0.17
PDCG T4T LT	0.29	0.25	PDCG PT4T LT	0.88	0.16
CGCG T4T HT	0.48	0.16	CGPD PT4T LT	0.93	0.14
PDPD PT4T HT	0.58	0.19	PDPD PT4T LT	0.96	0.09

First, as is seen in the overall proportion of each outcome, the behavior of the model varied across the experiment, differing from the behavior of human participants in particular ways across the 16 conditions. These results show that the behavior of model and participants was sensitive to both the game order and the confederate agent. The differences in the overall proportion also highlight differences in behavior between the model and participants. Second, conditions with the highest correlation are all conditions in which the confederate agent used the PT4T strategy. A higher correlation between model and human behavior when the confederate agent used the PT4T strategy would be expected, due to the fact that PT4T strategy is more apt to defect during a game, leading to less behavioral variability when the model or participant played with this strategy compared to the T4T strategy. Third, conditions with the lowest correlations occurred when the first game played was CG, suggesting that the model and participants behaved differently when playing CG. A difference in how the model and participant played the first game in a condition would have led to further difference between the model's predictions and the participants' behavior in the second game, due to between game learning. From these results, it can be concluded that the model's predictions could account for a certain degree of the human data and its ability to do so depended in part on the order games were played within a condition and the characteristics of the confederate agent.

### Repetition propensities

Table 5 shows that the majority of conditions where CG was the first game had the lowest correlations. These results suggest a difference in how the model and participants played CG, which would have led the model and human participants to adopt different strategies during the game. To compare the behavior of the model and participants, the round-by-round repetition propensity of the model and participants during CG when it was played first was calculated across all conditions. A difference score was then calculated by subtracting the model's round-by-round repetition propensity from the participant's. Difference scores at zero indicate that the repetition propensity of the humans and model were the same. A negative difference score indicates that the model under predicted the repetition propensity of participants and a positive difference score indicates that the model over predicted the repetition propensity of participants.

**Figure 6:**
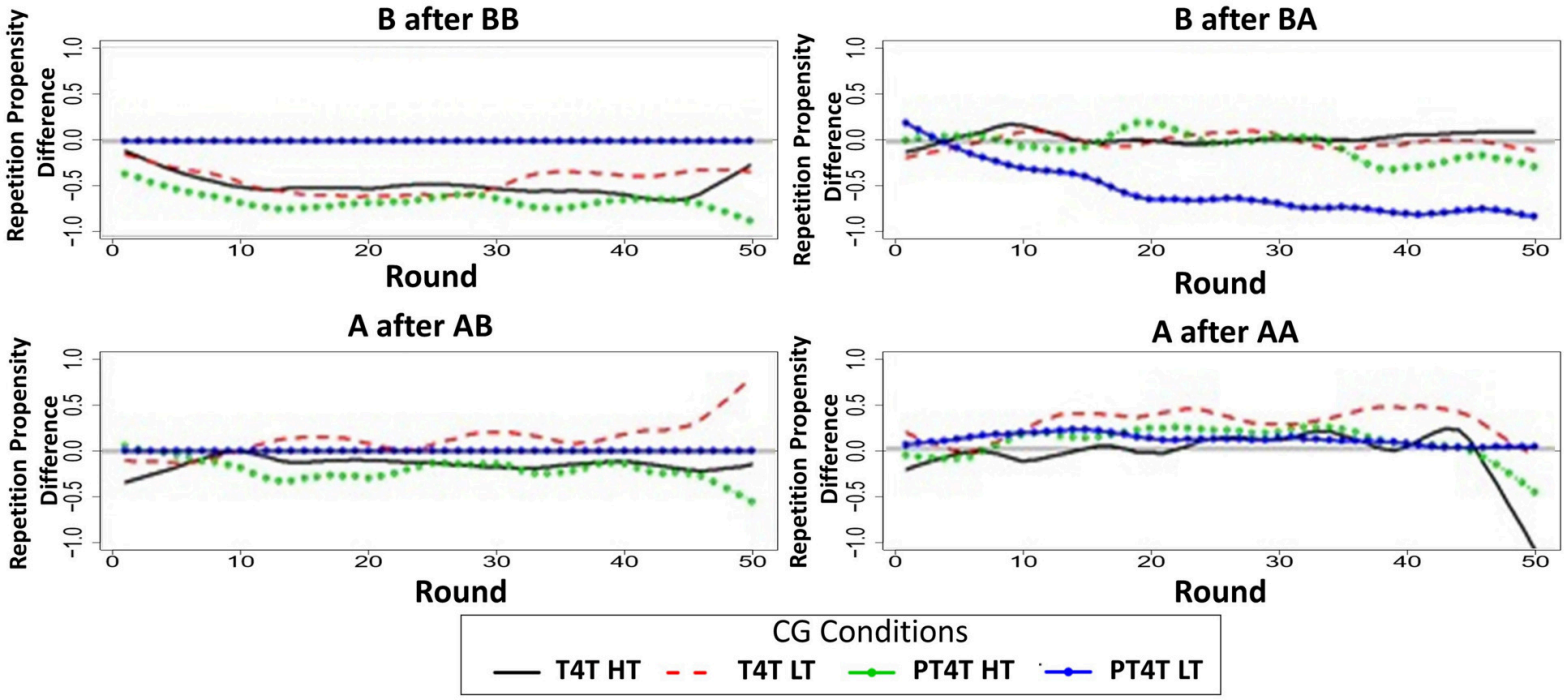
**The difference of the round-by-round repetition propensities between the model and participants for the four different repetition propensities during the Chicken Game (CG) in conditions when it was played first in the Tit-for-Tat (T4T) high trustworthiness (HT; solid black line), T4T low trustworthiness (LT; dashed red line), Pavlov-Tit-for-Tat (PT4T) HT (green line with sold dot), and PT4T LT condition (blue line with closed dot)**. All of the round-by-round repetition propensities were smoothed by using the loess function in *R* (smoothing parameter of 0.2), to remove some of the round-by-round variability.

Examining the differences in repetition propensity in CG^2^ (Figure 6), it becomes apparent that humans are more likely than the model to continue to cooperate after mutual cooperation (C after CC) and unilateral cooperation (C after CD) and continue to defect after unilateral defection (D after DC); in contrast, humans are less likely than the model to continue to defect after mutual defection (D after DD). This suggests that the current sample of participants may be more sensitive to payoff than the original sample. Recall that participants in the current study were paid for performance, whereas the participants in the original study (Juvina et al., 2013) were not. The payoff sensitivity may be stronger in CG because mutual defection is much more costly in CG than in PD. Thus, the poor predictive performance of the model in conditions that started with CG may be due to differences in how participants were incentivized in the two different studies and may not challenge the core assumptions of the model.

### Transfer effects

Previous research has found that, when PD and CG were played sequentially, specific between-game learning effects were seen, which the model was able to replicate (Juvina et al., 2015). However, it has yet to be shown if the model can accurately predict when between game learning effects (transfer effects) will and will not occur. In this study, deep transfer effects (defined above and more extensively in Juvina et al., 2013) could have occurred only in conditions where the confederate agent used the T4T strategy. Both optimal outcomes (i.e., mutual cooperation in PD and alternation in CG) can be achieved when playing with T4T, whereas only mutual cooperation can occur when playing with PT4T. Alternation cannot be achieved in CG when playing with PT4T, because PT4T does not reciprocate unilateral cooperation (i.e., it will continue to defect after a winning defection—win-stay). Thus, deep transfer effects could not have occurred in games with PT4T. All transfer effects were assessed using a paired *t*-test, run on the round-by-round proportion of an outcome when PD or CG was played first compared to the round-by-round proportion of the same outcome and game when played second game with a confederate agent who used the same strategy and trustworthiness (e.g., the proportion of the first 50 rounds of alternation during CG when played before PD, compared to the 50 rounds of CG played after PD, when playing with the T4T HT confederate agent). Finding that the proportion of an outcome occurred significantly higher when the game was played second indicates transfer of learning.

#### Deep transfer: PD to CG

**Figure 7:**
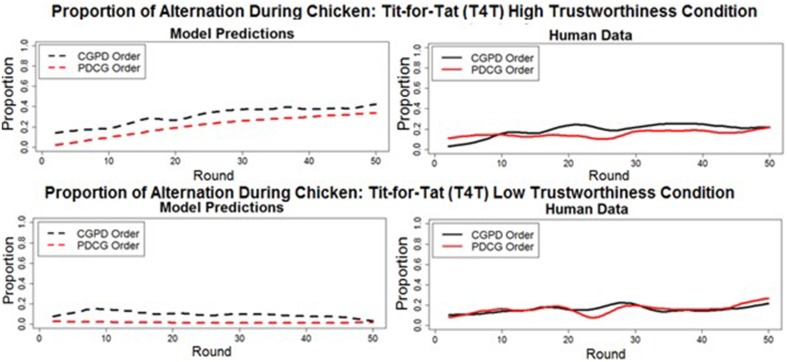
**The round-by-round proportion of alternation during Chicken Game in both the model predictions (dashed lines) and human data (solid lines), when played before (CGPD order) and after (PDCG order) the game Prisoner's Dilemma**. All of the overall round-by-round proportions were smoothed by using the loess function in *R*(smoothing parameter of 0.2), to remove some of the round-by-round variability.

A deep transfer of learning from PD to CG would consist of alternation occurring at a higher proportion when CG was played after PD (as observed in Juvina et al. (2013), in either the HT or LT T4T conditions. However, in the current study, in which human participants played games with simple preprogrammed agents, the model predicted that no deep transfer of learning from PD to CG would occur. Moreover, the model predicted that alternation would occur at a slightly lower proportion when CG is played after PD. Specifically, the model predicted that the proportion of alternation during CG when played before PD in the T4T HT (*M* = 31.5% *SD* = 10.09%) and LT (*M* = 9.9%, *SD* = 4.18%) conditions would decrease when played after PD, in both the T4T HT (*M* = 21.18%, *SD* = 10.7%) and the T4T LT (*M* = 1.7%, *SD* = 1.5%) conditions. The decrease in the proportion of alternation was found to be significant in both the T4T HT [*t*_(48)_ = 9.05, *p* < 0.001] and T4T LT [*t*_(48)_ = 14.41, *p* < 0.001] conditions. The lack of deep transfer from PD to CG was confirmed by the human data (Figure 7). The proportion of alternation significantly decreased during CG in the T4T HT condition from when played first (*M* = 19%, *SD* = 12%) to when played second [*M* = 15%, *SD* = 5.06%; *t*_(48)_ = 2.23, *p* < 0.03]. A slight decrease was seen in the proportion of alternation from when CG was played first in the T4T LT condition (*M* = 16.22%, *SD* = 7.39) to when it was played second (*M* = 15.71%, *SD* = 7.7%), but this was not found to be significant [*t*_(48)_ = 0.31, *p* > 0.05].

#### Deep transfer: CG to PD

A deep transfer of learning from CG to PD would consist of mutual cooperation occurring at a higher proportion in PD when played after CG compared to when played before CG. The model predicted that no deep transfer would occur between CG and PD in either the T4T HT or LT conditions. The proportion of mutual cooperation was predicted to decrease in the T4T HT condition, from when PD was played before CG (*M* = 49%, *SD* = 17%) to when PD was played after CG [*M* = 41%, *SD* = 18%; *t*_(49)_ = 3.94, *p* < 0.001]. A decrease in the proportion of mutual cooperation was also predicted to occur in the T4T LT condition, from when PD was played before CG (*M* = 11% *SD* = 0.05%), to when PD was played after CG [*M* = 3%, *SD* = 2%; *t*_(49)_ = 9.367, *p* < 0.001].

**Figure 8:**
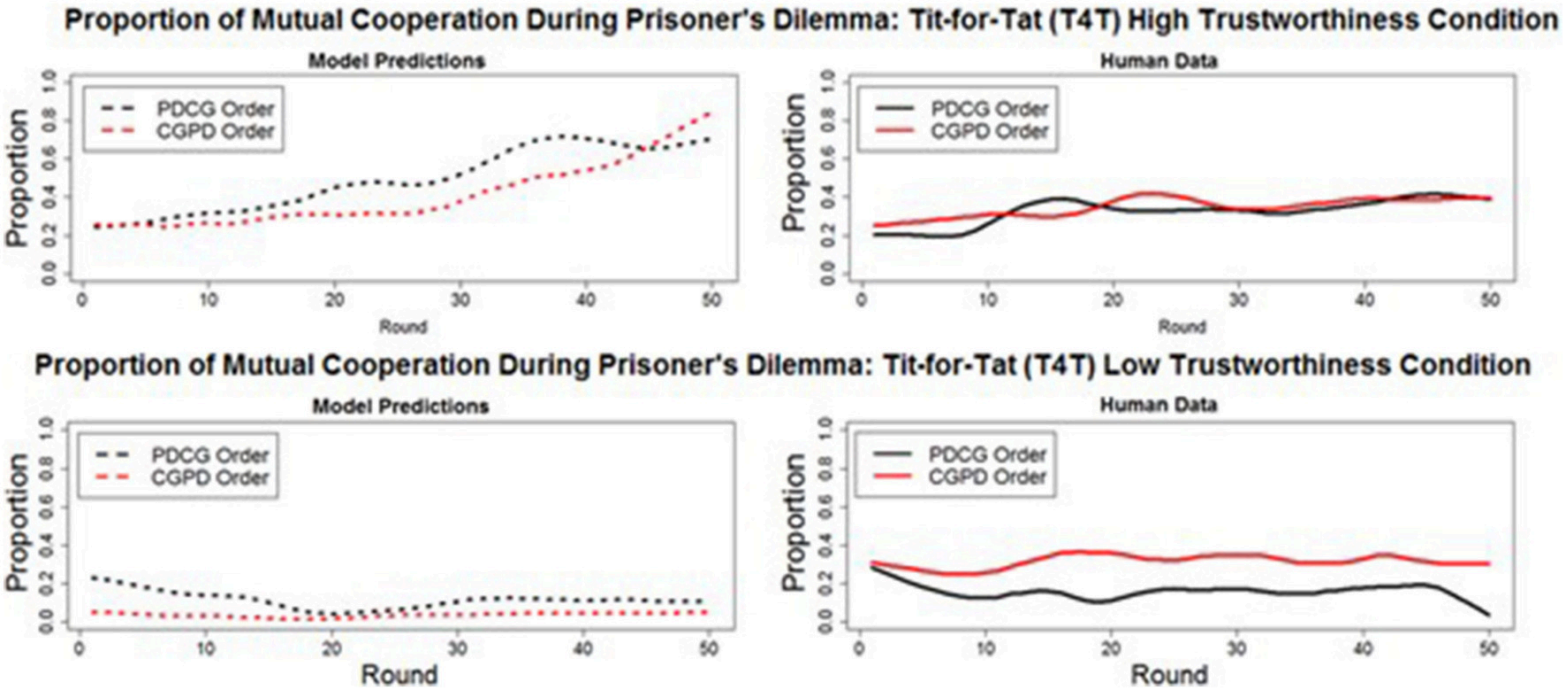
**The round-by-round proportion of mutual cooperation during Prisoner's Dilemma in both the model predictions (dashed lines) and human data (solid lines), when played before (PDCG order: black line) and after (CGPD Order: red line) the Chicken Game**. All of the overall round-by-round proportions were smoothed by using the loess function in *R* (smoothing parameter of 0.2), to remove some of the round-by-round variability.

**Figure 9:**
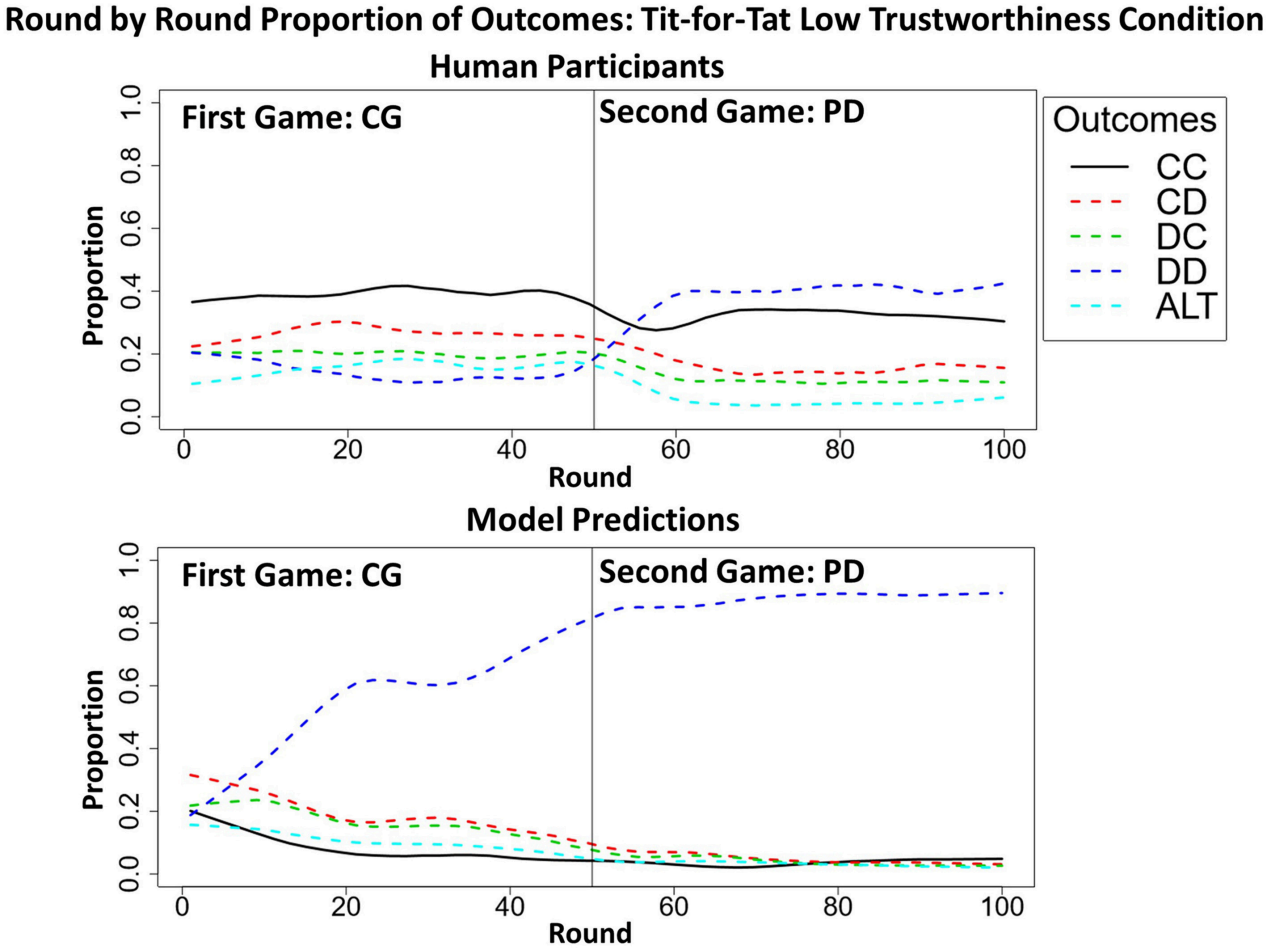
**The round-by-round proportion of all five outcomes [i.e., mutual cooperation (CC), unilateral cooperation (CD), unilateral defection (DC), mutual defection (DD), and alternation (ALT)] of both the model and participants during the CGPD Tit-for-Tat low trustworthiness condition, highlighting the surface transfer effect from CG to PD with the mutual cooperation outcome (thick black line) in the human data which was absent in the model's predictions**. All of the overall round-by-round proportions were smoothed by using the loess function in *R* (smoothing parameter of 0.2), to remove some of the round-by-round variability.

The human data confirm the model's prediction of the absence of deep transfer effects in the T4T HT conditions. A slight increase in the proportion of mutual cooperation in PD from when played before CG (*M* = 34%, *SD* = 8%) to when played after CG (*M* = 35%, *SD* = 6%) was found not to be significant [*t*_(49)_ = −1.3315, *p* > 0.05]. However, in the T4T LT condition, a significant difference was found in the proportion of mutual cooperation in PD from when played before CG (*M* = 16%, *SD* = 8%), to when played after CG [*M* = 30%, *SD* = 8%; *t*_(49)_ = −8.7, *p* < 0.001; Figure 8]. This is more likely to be a surface and not a deep transfer effect. When CG was played before PD in the T4T LT condition, the mutual cooperation outcome was chosen at a higher proportion than alternation and it might have transferred directly to PD (Figure 9). If there were a deep transfer effect, alternation would be expected to occur at a higher proportion than mutual cooperation in CG. In addition, mutual cooperation would be expected to increase from CG to PD, which was not observed.

The percentage of rounds in which mutual cooperation occurred during each game (CG and PD) in all 20 participants during the CGPD T4T LT condition was computed. A *t*-test revealed that the average proportion of mutual cooperation during CG (*M* = 19.60%, *SD* = 18%) was not significantly different than during PD [*M* = 15.45%, *SD* = 16.83%; *t*_(19)_ = 1.5713, *p* > 0.05] (Figure 9). These results suggest that transfer from CG to PD occurred along the games surface and not deep similarities. The model failed to predict this surface transfer effect.

### Model variants

The results presented above show that the human data matches the model's predictions to a certain degree. However, it is unknown how much the model's novel trust mechanism improved its ability to predict the participants' behavior. Juvina et al. (2015) found that the model's trust mechanism was necessary to account for the interaction between pairs of human participants. However, a human participant in this experiment interacted with a simple agent, not with another human. Did this modification render the proposed trust mechanism irrelevant to the new experimental paradigm? One observation suggesting that this may be the case is that no deep transfer effects occurred across the experimental conditions. Here, we examine this question in detail. The participants' responses to the state trust survey showed that their state trust was sensitive to characteristics of the confederate agent (i.e., strategy and trustworthiness, see Section Manipulation Check); however, it is unknown whether the participants used their trust in the confederate agent to inform their decisions. Due to the fact that the confederate agent used a simple strategy over the course of a game, informed only by the players' previous moves, perhaps a simpler model could have accounted as well or better for the behavior of human participants. To evaluate the necessity of the model's trust mechanism, we compared the results of the model's predictions to five model variants.

Each of the five model variants used the same learning mechanisms as the original model (i.e., instance based learning, sequence learning, and reinforcement learning), but lacked the trust mechanism, using only a single reward function to learn its strategy over the course of both games. The reward functions used in each of the variations of the model were the same used in Juvina et al. (2015), corresponding to the following reward functions: the model's payoff (P1_n_), the other player's payoff (P2_n_), the joint payoff of the model and the other player (P1_n_ + P2_n_), the model's payoff minus the payoff of the other player (P1_n_ − P2_n_), and the joint payoff of both players minus the previous payoff of the other player (P1_n_ + P2_n_ − P2_n−1_). Each of the five models was run in the 16 experimental conditions just as the original model.

We compared the overall *r* and RMSD between each of the five model variants and the human data against the *a priori* predictions of the original model. In addition, we calculated the number of individual conditions where a model variant fit the human data better than the original model (i.e., higher correlation and lower RMSD). The results show that the original model (including a trust mechanism) had both the highest *r* and the lowest RMSD compared to any other of the five model variations (Table 6). These results support the proposal that the trust mechanism is an important addition to the other learning mechanisms of the cognitive architecture, giving the model a principled way to change its strategy over the course of the game, even when playing with a scripted agent.

**Table 6:** **Correlation (*r*) and root mean squared deviation (RMSD) between models and human data for the original model (trust) and five model variants run in the same 16 conditions as the human participants**.

**Model**	***r***	**RMSD**	**Number of conditions**
Trust	0.66	0.19	NA
P1_n_	0.50	0.24	4
P1_n_ − P2_n_	0.46	0.30	1
P1_n_ + P2_n_ − P2_n−1_	0.32	0.26	2
P1_n_ + P2_n_	0.31	0.31	0
P2_n_	0.26	0.35	0

The third column (number of conditions) indicates the number of conditions in which a model variant fit the data better than the trust model.

### Necessity of the components of the trust mechanism

A comparison of the original model to five model variations revealed that a model which took into account trust was better able to account for human behavior than models which did not. However, this analysis does not reveal if each component of the model's trust mechanism (i.e., trust and trust invest accumulators) is necessary for the original model to account for the human data. Each of the model's accumulators represents a particular facet of trust, the trust accumulator representing the reactive component of trust and the trust invest accumulator representing the proactive component of trust. We propose that both components of the trust mechanism are necessary to account for human behavior. To examine the degree to which each component of the trust mechanism contributed to the model's predictions, we compared the original model's predictions (i.e., including both trust and trust invest accumulators), to a reactive model that used only the trust accumulator. The reactive model was run through all 16 conditions just as the original model, alternating between two payoff functions, attempting to maximize the joint payoff of both players minus the payoff of the other player's previous payoff (P1_*n*_ + P2_*n*_ − P2_*n*−1_), when the trust accumulator was greater than zero, or maximize its own payoff minus the payoff of the other player (P1_*n*_ − P2_*n*_), when the trust accumulator was at or below zero.

The reactive model's overall correlation was lower and RMSD higher between outcomes in each of the 16 conditions and human participants (*r* = 0.61, RMSD = 0.22), than were the same measures between the original model and the human participants (*r* = 0.66, RMSD = 0.19). A Steiger's *Z*-test revealed that the difference in the correlations was significant (*Z*_*H*_ = 13.86, *p* < 0.001). Thus, the addition of the trust invest accumulator significantly improves the model predictions. Additionally, a visual inspection of the results reveals that the reactive model generated predictions of behavior that were qualitatively different than the behavior observed in the human participants during the PT4T LT conditions.

**Figure 10:**
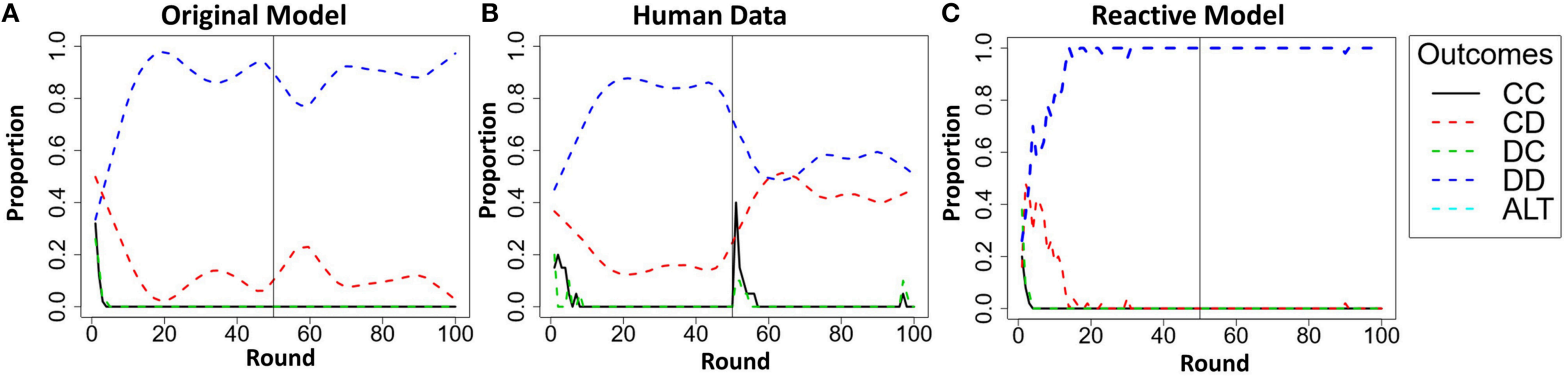
**Results of the original model predictions (A), human data (B), and reactive model (C) in the Pavlov-Tit-for-Tat Low Trustworthiness condition when playing Prisoner's dilemma (PD) and then Chicken Game (CG)**. Each graph shows the round-by-round proportion of each of the five possible outcomes, mutual cooperation (CC), unilateral cooperation (CD), unilateral defection (DC), mutual defection (DD), and asymmetric alternation (ALT). All of the overall round-by-round proportions were smoothed by using the loess function in *R* (smoothing parameter of 0.2), to remove some of the round-by-round variability.

In the PT4T LT conditions, the confederate agent will exploit unilateral cooperation by the model or the human participant. During these conditions the original model predicted a high proportion of mutual defection, but also predicted that participants would attempt to occasionally cooperate over the course of the game, which was also observed in the human data^3^. However, this type of behavior was not captured by the reactive model; instead, it predicted a high proportion of mutual defection and fewer attempts to unilaterally cooperate (Figure 10). The reactive model was limited by the fact that its behavior was solely reactive to the trustworthiness of the other player; once the confederate agent was shown to be untrustworthy, it would attempt to only maximize its own payoff for the remainder of both games.

**Figure 11:**
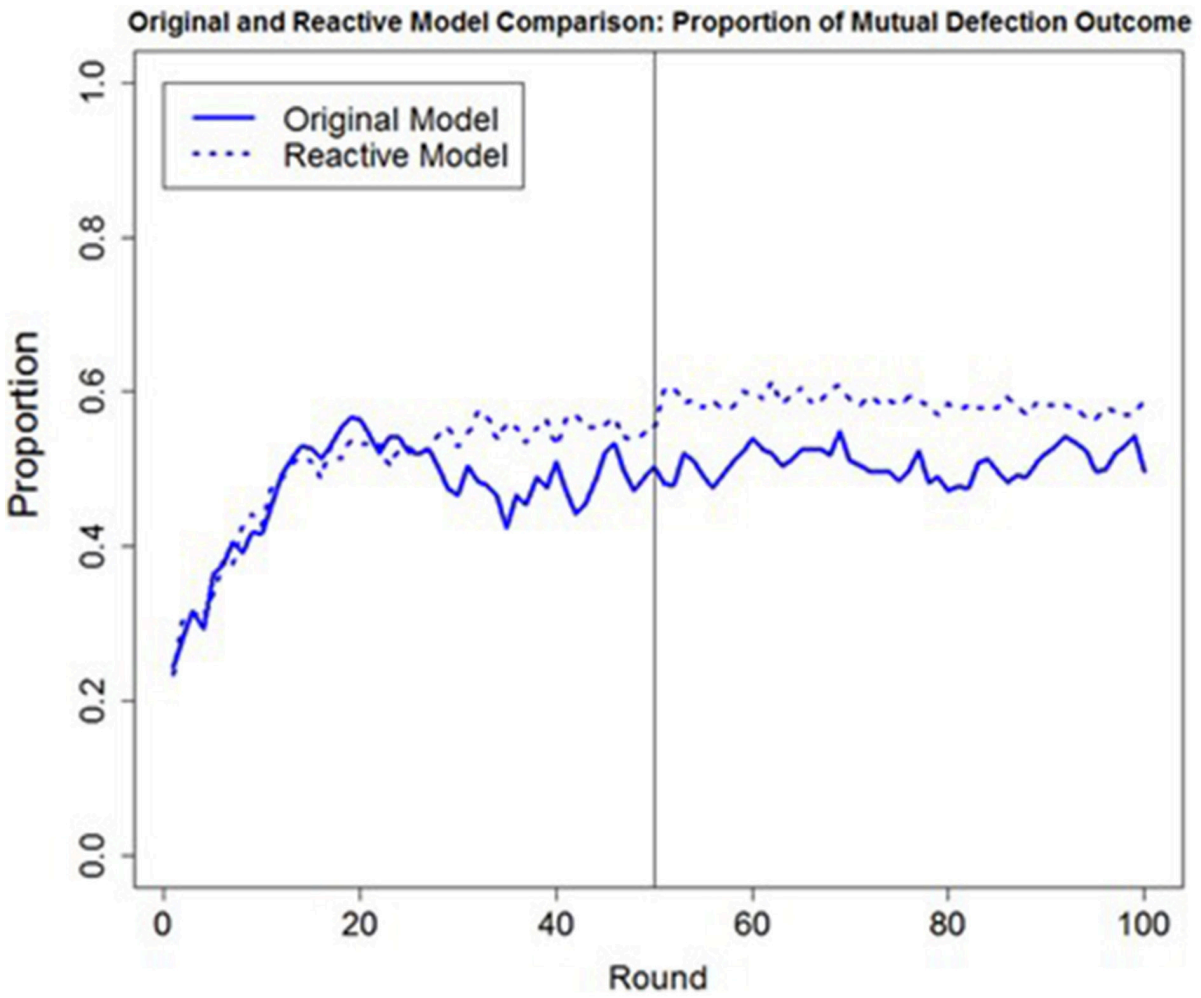
**The overall round-by-round proportion of mutual defection in the original trust model (solid blue line) that used both the trust and trust invest accumulator and the reactive model (dashed blue line) that used only the trust accumulator during a simulation of 16 different conditions where the confederate agent changed its strategy after the first game, changing from the Pavlov-Tit-for-Tat (PT4T) to the Tit-for-Tat (T4T) strategy or from the T4T to the PT4T strategy**.

One possible reason for only a small difference being observed between the *a priori* predictions of the original and reactive model was the fact that the confederate agent applied the same strategy and trustworthiness during both games. Had the confederate agent exhibited more variability in its behavior over the course of the games (e.g., changing its strategy or trustworthiness), we would predict a greater difference in the predictions between the original and the reactive model, due to fact that the reactive model would not be as sensitive to changes in the confederate agent's behavior. To examine this hypothesis, we ran the original and reactive models through a simulation of 16 different conditions, similar to the conditions presented in this experiment with the only difference being that the confederate agent changed its strategy after the first game (i.e., using the T4T strategy during the first game and then PT4T during the second game or vice versa). Although no human data are yet available to compare the two models' predictions, based only on simulations, the original model predicts less mutual defection than the reactive model across the 16 conditions (Figure 11). This difference in the predicted proportion of mutual defection suggests that the reactive model does indeed lack the ability to detect a change in the confederate agent's strategy from the first to the second game, making it less robust than the original model.

## Discussion

In this article, we examined the predictive validity of a computational cognitive model that was fit *post-hoc* to the aggregate behavior of individuals within and between two different games of strategic interaction, using how individuals are thought to develop trust in each other to inform their strategies (Juvina et al., 2015). Fitting a model to a dataset *post-hoc* leaves particular questions about the validity of the model unanswered. Here, we have attempted to test the model's predictive validity by addressing the question of whether the model could account for behavior of another sample of participants, playing under different conditions, and with specific strategies. We used the model to generate *a priori* predictions of human behavior across 16 different conditions, playing two games in various orders, and with a confederate agent playing specific strategies and having different levels of trustworthiness.

The model assumes that trust in the other player develops based on the dynamics of the interaction between the two players and, in turn, determines the strategy that the model adopts at any given time during a game. In line with the model's trust assumption, we hypothesized that participants' self-reported state trust (also referred to as learned or situational trust in the literature) would be sensitive to the manipulations of the confederate agent's strategy and trustworthiness. In contrast, we hypothesized that the participants' self-reported trait trust (also referred to as dispositional trust or trust propensity in the literature) would not be sensitive to the experimental manipulations. The results supported these hypotheses. Participants reported lower state trust in the confederate agent who used the PT4T compared to the T4T strategy and was low compared to high in trustworthiness, along being a significant predictor of their behavior during the second game. In contrast, the participants' reported trait trust was found to change over the course of the experiment and decrease slightly during the low trustworthiness conditions, but was not found to be a significant predictor of their behavior in the first game. The change reported in the participants' trait trust over the course of the experiment suggests that trait trust may change as a function of experience. Thus, the experimental manipulations were indeed related to trust and thus the current study provided a relevant test to a trust model.

The model's a priori predictions were partially corroborated; this is encouraging, considering the differences between the current and previous experimental designs. Each of the games was shortened from 200 to 50 rounds, which limited the experience of the participant with the confederate agent and could have affected the time available to learn the optimal outcome of a game and the time the participant had to develop trust in the other player. The participants played with a confederate agent that used a simple strategy and did not learn over the course of the game, whereas the model was originally fit to the aggregate results of pairs of human participants. In the current study, the participants were paid for performance, whereas in the original study they were paid a fixed amount. Any of these differences between the two experiments could have challenged the model to the point of catastrophic failure. An example of catastrophic failure for a trust model would be a case in which the empirical data were more accurately predicted by a simpler model that did not assume trust. We have compared the model's predictive performance to that of five model variants and showed that the trust mechanism of the original model caused a significant increase in prediction accuracy.

The trust mechanism allowed the model to switch strategies based on its experience with a particular player, its predictions of what the other player might do, and its assessment of the current game dynamics, something that none of the simpler model variants considered here had the ability to do^4^. Although each of the model variants used the same learning mechanisms as the trust model (i.e., IBL, sequence learning, and utility learning), they used the same payoff function throughout the entire game, which meant they couldn't change the strategy they learned. This led to cases in which the simpler models were taken advantage of, or unable to evade continual mutual defection. The trust model mitigated these problems by monitoring the trustworthiness of the other player and the need to build trust with the other player and switching strategies accordingly. The comparison of the trust model to the other model variants showed that the trust mechanism played an important role in accounting for human behavior over a wide range of conditions.

Additionally, evidence supporting the necessity of both components of the model's trust mechanism was found. A reactive model, using only the trust accumulator was found to account for human participants better than either of the model variants, but not as well as the original model that used the entire trust mechanism. The reactive model was limited by the fact that its trust in the confederate agent was governed solely by the trustworthiness of the other player. If the confederate agent was found untrustworthy, as in the PT4T LT conditions, the reactive model would begin to play selfishly and had no way to attempt to reestablish trust later on in the game. In comparison, human participants under these conditions were observed to occasionally attempt to cooperate over the course of the game despite evidence of untrustworthiness of the confederate agent. This behavior was predicted by the original model because of its trust invest accumulator, allowing it to better capture human behavior.

Finding that both of the trust accumulators used by the original model were necessary to account for human behavior lends support for the distinction between reactive and proactive facets of trust. Although the trust propensity and trustworthiness of another person (i.e., reactive components of trust) may be informative enough to base one's trust on in most situations, situations exist where the trustworthiness of another has been lost or is proven absent. In order to avoid continual mutually destructive outcomes, it may be necessary to proactively trust that the other person will become trustworthy, to rebuild a trust relationship. Although no large difference between the original and reactive model was found when explaining the behavior of human participants in the current experiment, we would predict a larger difference in the behavior of these two models under circumstances where the other player changes its behavior. Results from an unpublished simulation support this claim: when the confederate agent changed strategies during the second game, the original model was found to be more robust because it was able to detect the other player's change in strategy.

The authors would like to add a note of caution regarding interpretation of the results of this study as being characteristic of human–human interaction during these particular games. Others have noted differences in behavioral outcomes between humans playing in pairs with one another and humans playing with deterministic strategies (Harford and Solomon, 1967; Craig et al., 2013). Had the participants played in pairs with each other we would expect more variability in outcomes during the games and in the responses to the state trust questionnaire. However, the goal of this study was not to create confederate agents that mimicked human behavior, but to attempt to predict the response of participants to specific experimental manipulation (i.e., game order, strategy, and trustworthiness).

### Future research

The findings presented here suggest two possible future lines of research. The first is related to the sophistication of the players. Conceivably, the behavioral outcomes that occur when a human participant plays games of strategic interaction depend in part on the ability of the other player. Human participants who played PD and CG with other human participants exhibited deep transfer of learning effects (Juvina et al., 2013), whereas these effects were absent when participants played the same games with a scripted agent, as shown above. Craig et al. (2013) have suggested that, although having participants play games with simple predetermined strategies (e.g., T4T and Pavlov) allows for more experimental control during games of strategic interaction, these strategies may give rise to less interesting behavioral effects. We suggest that future research should examine the behavior of individuals when playing games of strategic interaction with more complex models, such as the one presented here.

The second line of future research is to further improve the ecological validity of the current model, by having it take into account non-verbal cues or facial expressions. For example, De Melo et al. (2011) found that when playing PD with a confederate agent that used the T4T strategy and displayed particular facial expressions during the game, the frequency of cooperation depended on the expressions that the agent displayed after certain outcomes. When given the opportunity, humans use more information than just previous choices and payoffs to inform their decision. Adding the ability to take in such features could improve the external validity of the model and its possible application to real world scenarios.

## Conclusion

In conclusion, a computational cognitive model incorporating a trust mechanism was able to generate more accurate predictions than simpler models that did not assume trust or used only part of the trust mechanism. The ability of the model to account for human-human interaction both within and between two different games, using no predetermined strategy, but instead learning to play and trust the other player over time was demonstrated elsewhere (Juvina et al., 2015). Finding that the same model also accounted for human behavior under a variety of new conditions suggests that the model incorporates plausible cognitive mechanisms explaining how humans develop and use trust in games of strategic interaction.

## Author contributions

MC implemented the experimental design, ran, analyzed the data, and wrote initial draft; IJ developed the experimental design, helped analyze data, and wrote the final manuscript; KG helped the planning the experiment, analyzing data, and was informative in writing and planning the early drafts.

### Conflict of interest statement

The authors declare that the research was conducted in the absence of any commercial or financial relationships that could be construed as a potential conflict of interest. The reviewer, Lars A. Bach, and handling Editor declared their shared affiliation, and the handling Editor states that the process nevertheless met the standards of a fair and objective review.

## Footnotes

^1^Qualtrics.com. the implementation platform used for this study, did not support random event permutations and so a pseudo-random variability design was implemented.

^2^The difference of the round by round repetition propensities during conditions that started with PD are not discussed here, but are reported in the Supplementary Material.

^3^We assume that participants occasionally cooperated over the course of the game in order to attempt to reestablish mutual cooperation between players. However, with only the behavioral outcomes we do not know the true motives of the participants' choices.

^4^We do not claim to have exhausted the full set of simpler models that could explain the empirical data.
