# Supplementary material for: Cognitive Model of Trust Dynamics Predicts Human Behavior within and between Two Games of Strategic Interaction with Computerized Confederate Agents
Source: Front Psychol. 2016 Feb 12;7:49. doi: 10.3389/fpsyg.2016.00049 (PMC4751270; doi:10.3389/fpsyg.2016.00049)
Supplement: Supplementary file 2 [file DataSheet2.docx]

Appendix A

**Trait Trust Questionnaire**

**^*^**Item was reverse coded

^a^Items came from Rotter (1967)

^b^Items came from Yamagishi (1986)

1. I generally have faith in humanity. ^a^

(1: Disagree very much) (2: Disagree slightly) (3: Neither agree nor disagree)

(4: Agree slightly) (5: Agree very much)

1. I feel that people are generally reliable.

(1: Disagree very much) (2: Disagree slightly) (3: Neither agree nor disagree) (4: Agree slightly) (5: Agree very much)

1. I generally trust other people unless they give me a reason not to.

(1: Disagree very much) (2: Disagree slightly) (3: Neither agree nor disagree) (4: Agree slightly) (5: Agree very much)

1. Most people are basically honest. ^b^

(1: Disagree very much) (2: Disagree slightly) (3: Neither agree nor disagree) (4: Agree slightly) (5: Agree very much)

1. Most people are trustworthy.

(1: Disagree very much) (2: Disagree slightly) (3: Neither agree nor disagree) (4: Agree slightly) (5: Agree very much)

1. Most people are basically good and kind.

(1: Disagree very much) (2: Disagree slightly) (3: Neither agree nor disagree) (4: Agree slightly) (5: Agree very much)

1. Most people are trustful of others.

(1: Disagree very much) (2: Disagree slightly) (3: Neither agree nor disagree) (4: Agree slightly) (5: Agree very much)

1. I am trustful.

(1: Disagree very much) (2: Disagree slightly) (3: Neither agree nor disagree) (4: Agree slightly) (5: Agree very much)

1. Most people will respond in kind when they are trusted by others.

(1: Disagree very much) (2: Disagree slightly) (3: Neither agree nor disagree) (4: Agree slightly) (5: Agree very much)

1. Hypocrisy is on the increase in our society.^a^

(1: Disagree very much) (2: Disagree slightly) (3: Neither agree nor disagree) (4: Agree slightly) (5: Agree very much)

1. One is better off being cautious when dealing with strangers until they have provided evidence that they are trustworthy.^a^

(1: Disagree very much) (2: Disagree slightly) (3: Neither agree nor disagree) (4: Agree slightly) (5: Agree very much)

1. Those devoted to unselfish causes are often exploited by others.^a^*

(1: Disagree very much) (2: Disagree slightly) (3: Neither agree nor disagree) (4: Agree slightly) (5: Agree very much)

1. Fear and social disgrace or punishment rather than conscience prevents most people from breaking the law.^a^

(1: Disagree very much) (2: Disagree slightly) (3: Neither agree nor disagree) (4: Agree slightly) (5: Agree very much)

1. Most experts can be relied upon to tell the truth about the limits of their knowledge.^a^

(1: Disagree very much) (2: Disagree slightly) (3: Neither agree nor disagree) (4: Agree slightly) (5: Agree very much)

1. Most people tell a lie when they can benefit by doing so. ^b^*

(1: Disagree very much) (2: Disagree slightly) (3: Neither agree nor disagree) (4: Agree slightly) (5: Agree very much)

1. The judiciary is a place where we can all get unbiased treatment.^a^

(1: Disagree very much) (2: Disagree slightly) (3: Neither agree nor disagree) (4: Agree slightly) (5: Agree very much)

1. Most people answer public opinion polls honestly.^a^

(1: Disagree very much) (2: Disagree slightly) (3: Neither agree nor disagree) (4: Agree slightly) (5: Agree very much)

1. Most repairmen will not overcharge, even if they think you are ignorant of their specialty.^a^

(1: Disagree very much) (2: Disagree slightly) (3: Neither agree nor disagree) (4: Agree slightly) (5: Agree very much)

1. Most people are primarily interested in their own welfare.^b^

(1: Disagree very much) (2: Disagree slightly) (3: Neither agree nor disagree) (4: Agree slightly) (5: Agree very much)

1. Most students in school would not cheat even if they were sure they could get away with it.^a^

(1: Disagree very much) (2: Disagree slightly) (3: Neither agree nor disagree) (4: Agree slightly) (5: Agree very much)

1. Most people can be counted on to do what they say they will do.^a^

(1: Disagree very much) (2: Disagree slightly) (3: Neither agree nor disagree) (4: Agree slightly) (5: Agree very much)

1. Most salesmen are honest in describing their products.^a^

(1: Disagree very much) (2: Disagree slightly) (3: Neither agree nor disagree) (4: Agree slightly) (5: Agree very much)

1. Most elected officials are really sincere in their campaign promises.^a^

(1: Disagree very much) (2: Disagree slightly) (3: Neither agree nor disagree) (4: Agree slightly) (5: Agree very much)

1. In these competitive times one has to be alert or someone is likely to take advantage of you. ^a^*

(1: Disagree very much) (2: Disagree slightly) (3: Neither agree nor disagree) (4: Agree slightly) (5: Agree very much)

Appendix B

^*^ Item was reverse coded

**State Trust Questionnaire**

1. I feel safe to take risks in this game knowing that the other player would not take advantage of me.

(1: Disagree very much) (2: Disagree slightly) (3: Neither agree nor disagree) (4: Agree slightly) (5: Agree very much)

1. The other player would not willingly undermine my earnings in this game.

(1: Disagree very much) (2: Disagree slightly) (3: Neither agree nor disagree) (4: Agree slightly) (5: Agree very much)

1. The other player behaves consistently.

(1: Disagree very much) (2: Disagree slightly) (3: Neither agree nor disagree) (4: Agree slightly) (5: Agree very much)

1. I believe that the other player wants to help me to make a good amount of payoff in this game.

(1: Disagree very much) (2: Disagree slightly) (3: Neither agree nor disagree) (4: Agree slightly) (5: Agree very much)

1. The other player can be trusted.

(1: Disagree very much) (2: Disagree slightly) (3: Neither agree nor disagree) (4: Agree slightly) (5: Agree very much)

1. The other player is trying to take advantage of me. *

(1: Disagree very much) (2: Disagree slightly) (3: Neither agree nor disagree) (4: Agree slightly) (5: Agree very much)

1. I feel that the other player is competent.

(1: Disagree very much) (2: Disagree slightly) (3: Neither agree nor disagree) (4: Agree slightly) (5: Agree very much)

1. The other player tries to make me lose in this game. *

(1: Disagree very much) (2: Disagree slightly) (3: Neither agree nor disagree) (4: Agree slightly) (5: Agree very much)

1. I believe that the other player is fair.

(1: Disagree very much) (2: Disagree slightly) (3: Neither agree nor disagree) (4: Agree slightly) (5: Agree very much)

1. I would not let the other player have any influence over my payoff. *

(1: Disagree very much) (2: Disagree slightly) (3: Neither agree nor disagree) (4: Agree slightly) (5: Agree very much)

1. I would be willing to let the other player have complete control over the outcomes of this game.

(1: Disagree very much) (2: Disagree slightly) (3: Neither agree nor disagree) (4: Agree slightly) (5: Agree very much)

1. I understand the reasoning behind the other players moves.

(1: Disagree very much) (2: Disagree slightly) (3: Neither agree nor disagree) (4: Agree slightly) (5: Agree very much)

1. I know in advance what moves the other player will make.

(1: Disagree very much) (2: Disagree slightly) (3: Neither agree nor disagree) (4: Agree slightly) (5: Agree very much)

1. I like playing with the other player in this game.

(1: Disagree very much) (2: Disagree slightly) (3: Neither agree nor disagree) (4: Agree slightly) (5: Agree very much)
