# Supplementary figures and images for: Cognitive Model of Trust Dynamics Predicts Human Behavior within and between Two Games of Strategic Interaction with Computerized Confederate Agents
Source: Front Psychol. 2016 Feb 12;7:49. doi: 10.3389/fpsyg.2016.00049 (PMC4751270; doi:10.3389/fpsyg.2016.00049)

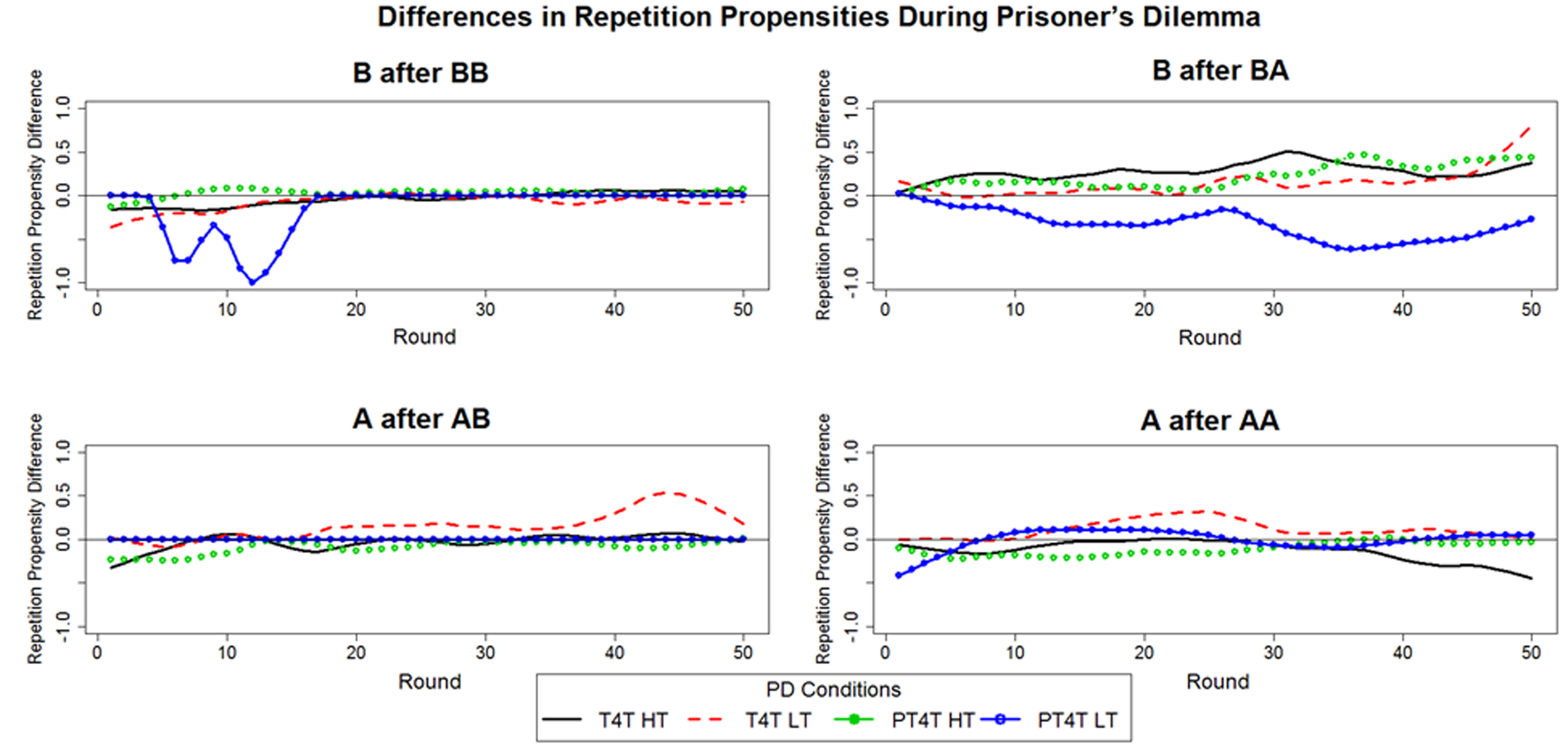

Supplement: Supplementary file 3 [file Image1.JPEG]
